# Angiosarcoma Arising in a Patient with a 10-Year-Old Hemangioma

**DOI:** 10.1155/2014/185323

**Published:** 2014-12-31

**Authors:** Michael J. Nathenson, Diana Molavi, Albert Aboulafia

**Affiliations:** ^1^University of Maryland Greenebaum Cancer Center, 22 South Greene Street, Suite 9D10, Baltimore, MD 21201, USA; ^2^Department of Pathology, Sinai Hospital, 2401 W. Belvedere Avenue, Baltimore, MD 21215, USA; ^3^Sarcoma Services, Cancer Institute Sinai Hospital, 2401 W. Belvedere Avenue, Baltimore, MD 21215, USA; ^4^Department of Orthopaedics, University of Maryland Medical Center, 22 South Greene Street, Baltimore, MD 21201, USA; ^5^Harry and Jeanette Weinberg Cancer Institute, MedStar Franklin Square Medical Center, Baltimore, MD 21237, USA

## Abstract

The transformation of a benign hemangioma into a malignant angiosarcoma has been rarely reported, with only 11 cases reported in the literature. There have been no reports of malignant transformation of hemangioma into angiosarcoma in association with epithelioid hemangioendothelioma, to our knowledge. The existence of precursor malignancies in the tumorigenesis of sarcomas is still not clearly defined. We describe the case of a 40-year-old woman with a preceding history of a suspected hemangioma for ten years, who upon resection was found on histology to have evidence of a hemangioma with an associated area of epithelioid hemangioendothelioma as well as areas of overt high grade epithelioid angiosarcoma. These findings raise the possibility of the evolution of hemangioma to epithelioid hemangioendothelioma, and the latter to overt angiosarcoma. The patient was managed as having a high grade sarcoma with wide resection and radiation. She declined systemic adjuvant chemotherapy after a thorough discussion about the risks and benefits of chemotherapy, and she currently remains disease free one year after the surgery.

## 1. Background

Soft tissue sarcomas are rare malignant neoplasms comprising <1% of all adult malignancies [1]. There are approximately 50 different subtypes of soft tissue sarcomas, making the study of this group of neoplasms particularly challenging. It is, therefore, not surprising that much of the pathogenesis of these tumors is still unknown. In carcinomas, the concept of adenomas as a precursor to invasive carcinomas is generally accepted, such as in tubular adenoma, a precursor lesion to invasive colon carcinoma. The role of precursor malignancies has yet to be fully determined in soft tissue sarcomas, with the exceptions of progression of neurofibroma to malignant peripheral nerve sheath tumor or lipoma into dedifferentiated liposarcoma. There are examples of precursor malignancies for bone sarcomas such as osteochondroma or enchondroma transforming to chondrosarcoma.

In the case of malignant vascular tumors such as angiosarcoma, there have been several case reports of angiosarcomas arising at the site of irradiated hemangiomas [2, 3]. There have been only 11 cases reported of angiosarcoma arising in the setting of hemangiomas without any preceding radiation [4–11]. None of these previous reports have established the latency period between the development of a benign hemangioma and the transformation to a malignant angiosarcoma. We describe a case report of a 40-year-old woman with a history of a hemangioma for ten years that, upon resection for symptomatic reasons, was found to have a component of epithelioid hemangioendothelioma as well as epithelioid angiosarcoma. This suggests that some hemangiomas despite long periods of latency may have the potential to transform into more malignant lesions.

## 2. Case Presentation

A 40-year-old woman was referred to The Orthopaedic Oncology Center with a chief complaint of “pain in my left calf for the past ten years.” She reported that her symptoms of pain in her calf began approximately 10 years ago. She described the pain initially as throbbing in nature that was exacerbated with activity and with ankle dorsiflexion and was relieved with rest and warm compresses. At that time, she noted a mass in her left calf that was about 2 inches in size, which did not fluctuate in size and was nontender. She continued to have mild to moderate pain on a regular basis but did not seek medical attention. Instead, she avoided prolonged walking. Approximately three years ago, the pain became more constant and severe. She began walking with a limp due to the inability to dorsiflex her foot to neutral position and she walked on the tip toes of her left foot. For the past 3 years, she has only been able to walk on her tip toes due to an equinus contracture. Three months prior to presentation, in our office, the patient experienced an acute exacerbation of pain in her left calf and sought medical attention from her primary physician. She was prescribed narcotic analgesia without significant relief of her symptoms. The patient was subsequently referred for an MRI. As initial work-up for this lesion, she had an MRI of the left lower extremity.

**Figure 1:**
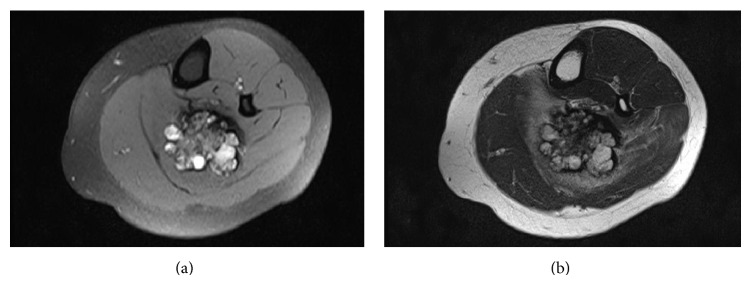
T1 weighted fat suppressed (a) and T2 (b) images of the left lower extremity, with no intravenous contrast, showing a deep-seat posterior ankle well-circumscribed ovoid mass centered in the soleus muscles measuring 6.8 cm in the craniocaudal dimension, 5.2 cm in the transverse oblique dimension, and 4.6 cm in the oblique dimension, with lobulations and septations, as well as heterogeneous signal intensity on T1 and T2 weight images, suggestive of a hemangioma.

This showed an ovoid mass centered in the soleus muscle measuring 6.8 cm in the craniocaudal dimension, 5.2 cm in the transverse oblique dimension, and 4.6 cm in the oblique dimension. Edema in the soleus muscle and surrounding musculature were noted (Figure 1).

Following the MRI, she was referred to the orthopaedic oncology service. She believed that the mass had increased slightly from the time she first noticed it 10 years earlier. She had no other associated symptoms. Her past medical history included only a previous hysterectomy with tubal ligation and unilateral oophorectomy and gastroesophageal reflux. She had no history of prior radiation therapy. The only medication she was taking was low dose narcotics for her pain. Her only allergy was a rash to nitrofurantoin. She was married and denied any alcohol, tobacco, or illegal drug use. She had no family history of sarcomas, only a grandmother with non-small cell lung cancer and a paternal aunt and uncle with adenocarcinoma of the colon and an aunt with uterine cancer. Her physical exam revealed an equinus contracture of 20 degrees with plantar flexion from 20–30 degrees of the left ankle. There was a well-defined, firm, and tender deep-seated 5 × 5 cm soft tissue mass in the midcalf. There were no cutaneous changes or skin discoloration or lymphadenopathy. The mass did not change in size when the extremity was elevated or placed in a dependent position. Though this lesion was suspected to be a benign cavernous hemangioma, due to the patient's significant symptoms, she elected for the resection of her left lower extremity soft tissue tumor.

At the time of surgery, a longitudinal incision along the posteromedial aspect of the left leg was made. The plane between the gastrocnemius and soleus was developed and the mass was easily identified. Grossly, there was an irregular red-brown to tan-appearing friable mass intertwined with skeletal muscle with associated large vascular channels consistent with a cavernous hemangioma. Manipulation of the ankle under anesthesia was performed bringing the ankle to 10 degrees of dorsiflexion in order to correct the equinus contracture. She was placed in an ankle dorsiflexion splint postoperatively.

## 3. Pathology

### 3.1. Gross Description

The pathology specimen consisted of multiple irregular fragments of soft tissue including skeletal muscle. Sectioning revealed a poorly defined, hemorrhagic, and red-brown lesion measuring at least 5 cm in the greatest dimension. Multiple cystic spaces with blood clot suggestive of blood vessels were noted.

### 3.2. Microscopic Description

**Figure 2:**
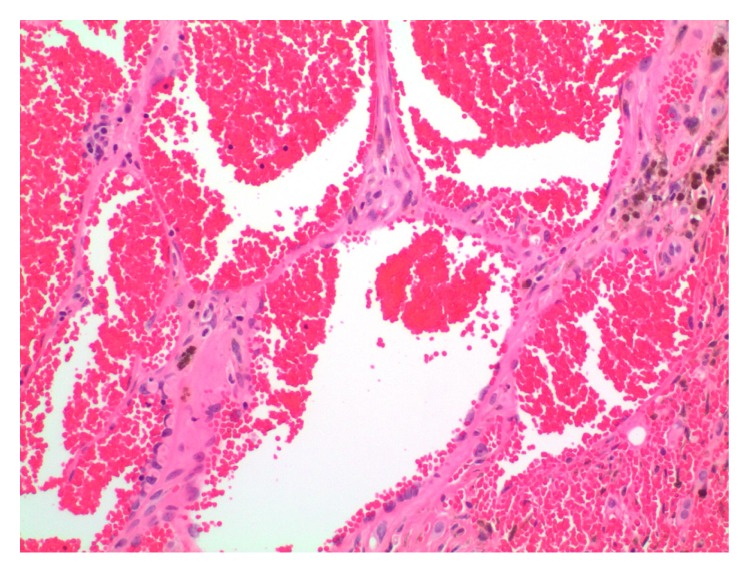
An area indistinguishable from cavernous hemangioma with large blood-filled spaces lined by flattened bland endothelium.

**Figure 3:**
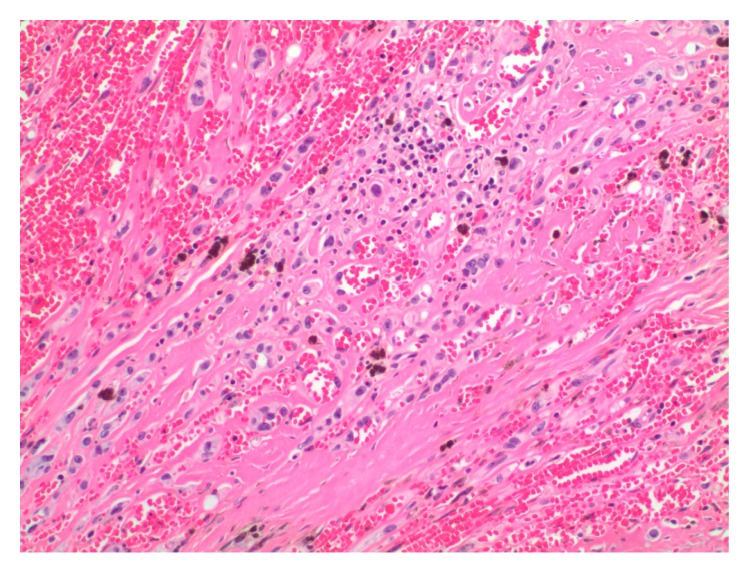
An area more typical of epithelioid hemangioendothelioma, a low-grade malignancy, with cords of tumor cells in a hyalinized stroma. Some cells show intracytoplasmic vacuoles with red cells, suggestive of primitive vascular spaces.

**Figure 4:**
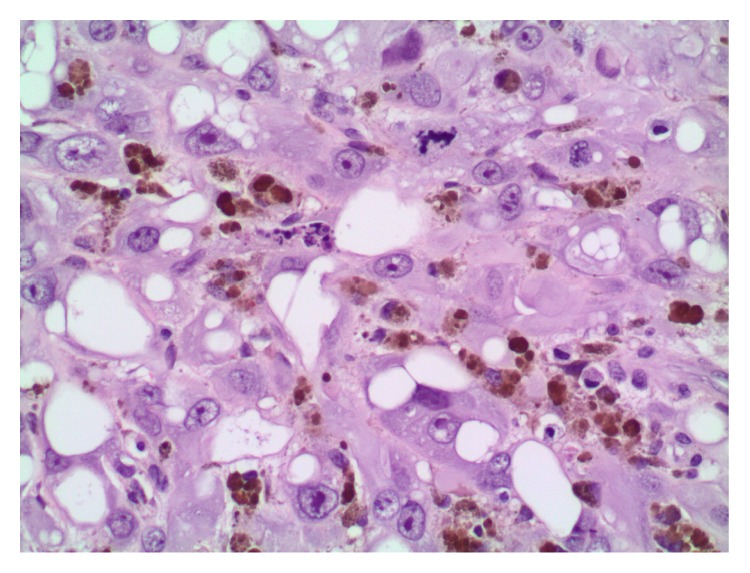
A well-circumscribed area of high grade angiosarcoma. The tumor cells are large and polygonal with abundant cytoplasm, which corresponds to the epithelioid variant. The nuclei are very atypical and pleomorphic, with large nucleoli and irregular nuclear outlines; abnormal mitoses are present. The abundant pigment and prominent nucleoli led to the consideration of a melanoma; however, melanoma markers were negative, and the pigment is hemosiderin.

On sectioning, the majority of the lesion was indistinguishable from a cavernous hemangioma with large blood-filled spaces lined by flattened bland endothelium (Figure 2). There were smaller areas more typical of epithelioid hemangioendothelioma and a low-grade malignancy, present as well. These were exemplified by cords of tumor cells in a hyalinized stroma. Some cells show intracytoplasmic vacuoles with red cells, suggestive of primitive vascular spaces (Figure 3). In addition, there was a small well-circumscribed area of high grade angiosarcoma. The tumor cells were large and polygonal with abundant cytoplasm, which corresponds to the epithelioid variant. The nuclei were very atypical and pleomorphic, with large nucleoli and irregular nuclear outlines; abnormal mitoses were present (Figure 4).

### 3.3. Diagnosis

Epithelioid angiosarcoma, apparently arising in a 10-year-old hemangioma. Some areas are indistinguishable from epithelioid hemangioendothelioma, but other foci show high grade epithelioid angiosarcoma.

### 3.4. Immunohistochemical Findings

**Figure 5:**
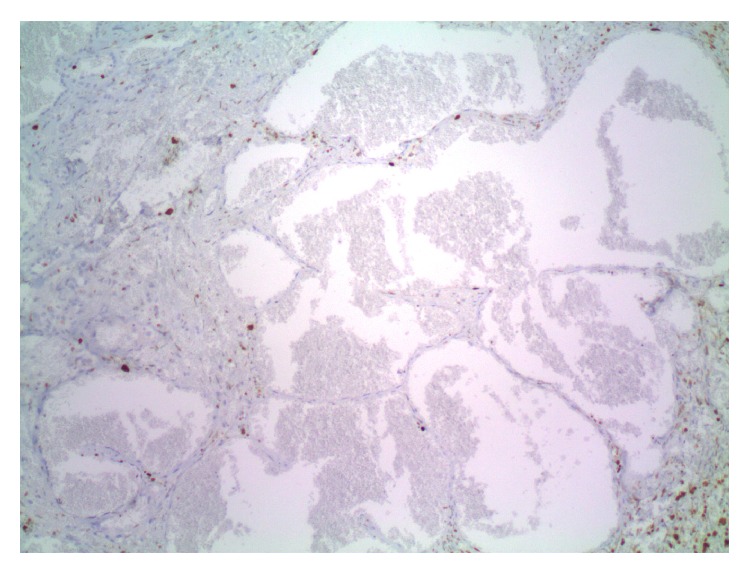
Cavernous hemangioma with virtually no Ki67 staining.

**Figure 6:**
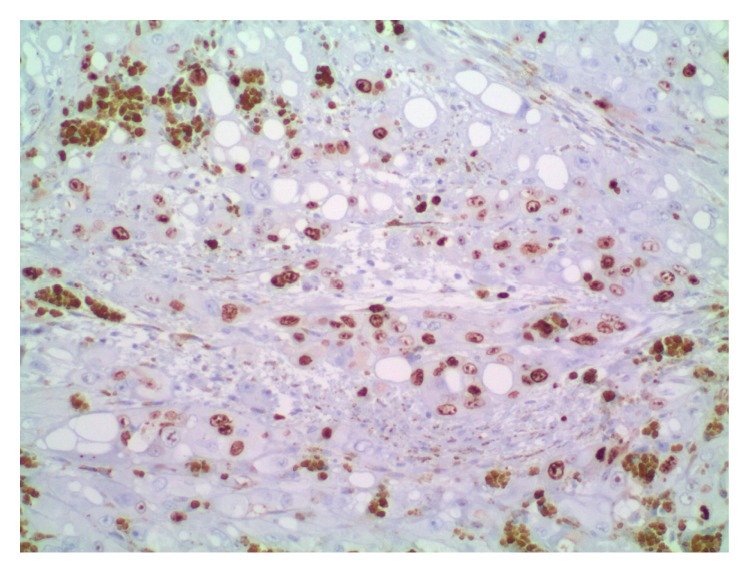
Area of angiosarcoma with extensive Ki67 staining.

Immunohistochemical stains show that the lesion expresses CD31, consistent with a vascular neoplasm. Pankeratin, S100, desmin, and HMB45 are negative. There was virtually no Ki67 staining of the hemangioma-like areas (Figure 5); however, there was extensive Ki67 staining of the angiosarcoma area (Figure 6).

Once the patient was diagnosed with angiosarcoma, she underwent a noncontrast CT of the chest that did not show any evidence of pulmonary nodules. She then proceeded to a wide local reexcision which did not show any residual tumor. She received adjuvant radiation therapy, but she elected to decline adjuvant chemotherapy after discussion of the risks and benefits. She is currently alive and free of disease recurrence, one year after the surgery.

## 4. Discussion

The tumorigenesis in sarcomas is still debated, compared to tumorigenesis in carcinomas where precursor malignancies are well understood. Most angiosarcomas arise de novo. Since the 1970s, there have been only four case reports and two case series, comprising 11 cases, describing angiosarcomas (AS) arising from benign hemangiomas in the absence of irradiation, suggesting that, for this subtype of soft tissue sarcoma, the pathogenesis may follow the malignant transformation of a benign lesion in some cases [4–11]. However, many of these cases are not convincing. For example, in 1970, Girard et al. [4] reported three cases of low-grade cutaneous angiosarcoma arising from port-wine stains in children; some, that is, Fletcher et al., [5] would argue that these cases represent capillary hemangiomas in the cellular stage and not true angiosarcomas. In addition, two of the cases reported by McRae et al. [6] and Chalet et al. [10] occurred after resection of the initial benign lesions. The remaining six cases reported by Mandahl et al., [7] Tohme et al. [8, 9], and Rossi and Fletcher [11] are more convincing. Particularly, in 2002, Rossi and Fletcher [11] described four cases of newly diagnosed angiosarcoma, with preceding duration of 1–24 months. All four of these cases had two distinct components on histology, one malignant consistent with angiosarcoma, and the other benign consistent with arteriovenous or capillary hemangiomas. One possible explanation for these case reports is the transformation of a benign hemangioma into a malignant angiosarcoma. However, these previous reports have not established a latency period between the development of benign and malignant lesions nor have any suggested a low-grade malignancy as an intermediate step in the transformation to angiosarcoma.

We describe a case of a patient with a presumed hemangioma for ten years preceding her diagnosis of angiosarcoma. Her pathology shows areas of benign hemangioma and epithelioid hemangioendothelioma and a focus of high grade epithelioid angiosarcoma. There are two possible explanations for this pathologic result. The first possibility is the malignant transformation of a hemangioma to angiosarcoma, with an intermediate step of low-grade malignancy. The second possibility is a composite hemangioendothelioma. This rare entity was first described by in 2000 by Nayler et al. [12]. So far, there have been approximately 30 cases of composite hemangioendothelioma reported in the literature [13]. This lesion occurs most frequently on the extremities but has been reported in the head and neck and kidney and spleen as well [14, 15]. This lesion is usually composed of a mixture of epithelioid hemangioendothelioma, retiform hemangioendothelioma, spindle cell hemangioendothelioma, and low-grade angiosarcoma-like areas [12]. However, our case is composed mostly of benign hemangioma with smaller areas of epithelioid hemangioendothelioma and an area of high grade angiosarcoma. This does not correlate with the prior descriptions of composite hemangioendotheliomas, which have only described areas of low-grade angiosarcoma [12]. In addition, it is currently unclear whether a composite hemangioendothelioma arises de novo or as a step-wise process from a precursor lesion.

Therefore, our case likely represents the transformation of a benign hemangioma or low-grade hemangioendothelioma which had been present for 10 years into a high grade angiosarcoma. This supports the hypothesis that benign vascular tumors can undergo malignant transformation and argues for increased surveillance in those patients with a previous diagnosis of a benign hemangioma that have changing symptoms or size of their lesion.

## 5. Conclusion

In summary, we describe a case of a 40-year-old woman with a ten-year history of a hemangioma that upon resection showed an area of transformation into high grade angiosarcoma, supporting the hypothesis that benign hemangioma can transform into more malignant tumors.
